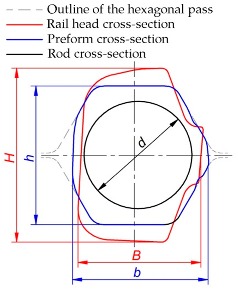# Skew Rolling of Rods from Scrap Rail Heads

**DOI:** 10.3390/ma12182970

**Published:** 2019-09-12

**Authors:** Janusz Tomczak, Zbigniew Pater, Tomasz Bulzak

**Affiliations:** Faculty of Mechanical Engineering, Lublin University of Technology, 36 Nadbystrzycka Str., 20-618 Lublin, Poland

**Keywords:** helical roll pass, skew rolling, railway rail, rod rolling

## Abstract

This paper presents the results of theoretical and experimental investigations of a new process of rolling rods from scrap rail heads. First, the industrial applications of scrap railway rails and methods of their recycling are discussed, and then the concept of two-stage rolling of rods from heads cut off from scrap rails is proposed. In the first stage of the process, a rail head preform was rolled in a hexagonal pass of a longitudinal rolling mill. Then in the second stage, the hexagonal bar was skew rolled into a rod in a helical roll pass. Theoretical considerations were based on finite element numerical modelling. The rolling process was simulated under 3D deformation using Forge NxT v.1.1 software developed by Transvalor Company. Calculations were carried out to determine the material flow kinematics, strength, and thermal parameters of the process and to identify the phenomena that might constrain its implementation. The numerical results were verified in experimental tests, during which preforms and rods were formed from scrap rail heads. The tests were conducted in longitudinal and skew rolling mills. The results indicate that rods can be effectively formed from scrap rail heads in just two steps. Rods obtained using the proposed method can be used as full-featured, semifinished products for the manufacture of various types of machine parts.

## 1. Introduction

The large supply of used railway rails has spurred the search for effective methods of managing and processing railway scrap. At present, scrap rails are recycled in two ways: they are either founded in steelworks [1,2], or, alternatively, selected rail sections are used as stock material for forging or rolling [3,4]. In the case of traditional recycling of scrap railway rails, 80–90% of the total cost of processing 1 Mg of rails goes to the steelmaking process, in which scrap metal is processed back into steel, and 10–20% of the cost is associated with forming the recovered steel into a finished product (e.g., rolling of bars, rods, sheet metal, strips, etc.) [5,6]. Given this, it seems reasonable to eliminate the steelmaking process from the recycling procedure and to produce full-featured products directly from used railway rails. What also supports this approach are the very good properties of the steel from which the rails are made [7,8]. The high strength parameters of railway steel make it a good choice for the production of such elements as reinforcing bars, rods used for the production of springs, rods for rolling grinding mill balls, and many other products and semi-finished products commonly made from high-strength steel. Currently, in most cases, reprocessing is performed using rail heads cut off from worn rails, as they have the most compact shape [9]. However, because of their irregular cross-sections, the use of scrap rail heads is limited. They are mainly utilized as billets in die forging of grinding mill balls. Unfortunately, this technology is inefficient and burdened with large energy losses as well as generating a substantial amount of flash. A much more effective way of reprocessing rail heads is to roll them into rods, bypassing the steelmaking process. 

At present, longitudinal rolling with the use of grooved rolls is one of the most efficient and widely used rolling methods for producing steel bars. Such bars are rolled in metallurgical conditions from steel blooms produced by continuous casting processes [10]. Depending on the required bar diameter and quality, longitudinal rolling processes are performed with the use of up to several roll passes. Rolling mill configurations depend on the required production rate. The most popular rolling configurations applied in the longitudinal rolling of bars include a reversing configuration, which is less efficient yet requires the use of fewer mill stands, or a highly efficient continuous configuration, in which the number of employed mill stands is equal to the number of roll passes. The rolling of bars is seldom performed in skew rolling mills [11,12]. This mainly results from the fact that skew rolling is less efficient than longitudinal rolling; it is characterized by faster tool wear and the risk of cavity formation in the center of the cylindrical billet (the so-called Mannesmann effect) [13]. As a result, this method is primarily employed in the production of seamless tubes [14], in which skew rolling mills are used for piercing [15] as well as diameter reduction and elongation [16]. Skew rolling processes are predominantly used for rolling bars from hard-to-deform materials such as alloys of titanium [17,18], nickel, and magnesium [19]. The skew rolling method ensures high accuracy of products and, thus, is widely used for straightening and sizing longitudinally rolled bars in order to improve their quality [20].

**Figure 1:**
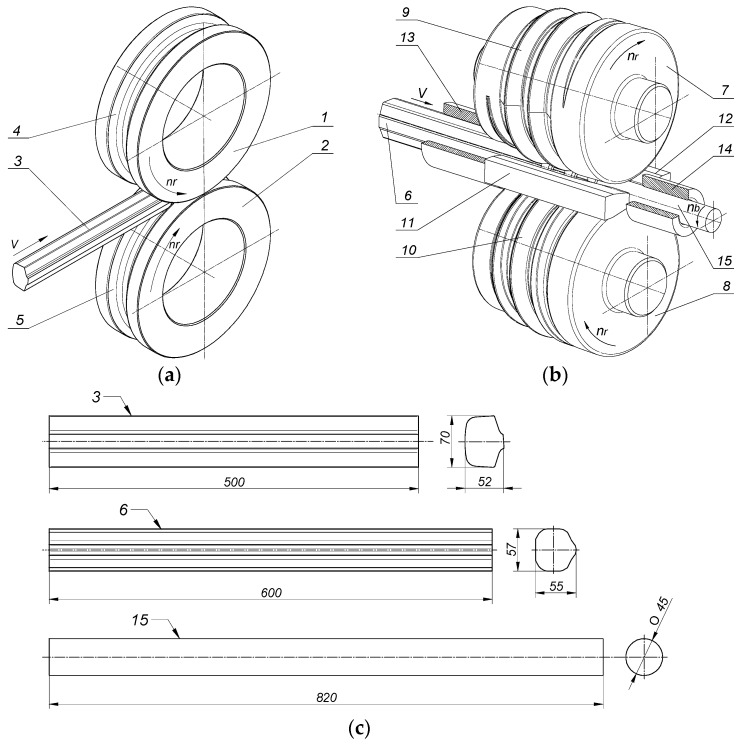
Schematic of rod rolling from scrap rail heads, with the dimension in mm: (**a**) rolling of a preform in a longitudinal mill; (**b**) rolling of a rod in a skew mill; (**c**) shapes of the blanks after the successive passes; 1,2—grooved rolls of the longitudinal mill; 3—rail head; 4,5—grooves forming a hexagonal pass; 6—blank with a nearly regular polygonal cross-section rolled in the longitudinal mill; 7,8—helical rolls; 9,10—helical wedge collars; 11,12—guides; 13—guiding sleeve; 14—discharge sleeve; 15—final rod product.

According to literature reports, Ukrainian railways have been planning to establish special plants for rolling scrap rail heads into rods with diameters from *ϕ* 32 to *ϕ* 6.5 mm [21]. The technology they want to use is based on longitudinal rolling, in which a system for calibrating groove passes allows to produce *ϕ* 32 mm rods in seven passes and *ϕ* 6.5 mm rods in 20 passes. To implement this project, the company would have to build several rolling mills and equip them with as many tool sets as there are passes to be performed. This means that the proposed technology would require large investments. Moreover, in a rolling system of this type, the maximum diameter of the rods that can be formed is relatively small compared to the cross-section of a worn rail head. Also, the rod diameters that can be obtained depend on the previously designed (nonadjustable) passes. Considering the limitations of longitudinal rolling of rods from scrap rail heads, we propose a new technology for rolling rods (Figure 1), which is implemented in only two stages (two passes) [22,23]. In the first stage, heads cut off from worn rails are compressed in a hexagonal pass of a longitudinal rolling mill (Figure 1a). This is done to obtain a relatively regular cross-sectional shape of the preform. Then, the nearly regular polygon preforms are rolled into rods in a two-roll skew rolling mill using helical passes (Figure 1b). In this solution, rods with a wide range of diameters can be rolled in only two passes (Figure 1c). Rod diameter will depend solely on the position of the tools in the skew rolling mill, and not on the geometry of the rolls used, as is the case with traditional longitudinal rolling processes. Skew rolling also allows to obtain a good strain distribution, thus improving the strength properties of the rolled material compared to longitudinal rolling. The basic parameters of rolling *ϕ* 45 mm diameter rods from scrap rail heads in a longitudinal and a skew rolling mill are compared in Table 1.

**Figure 2:**
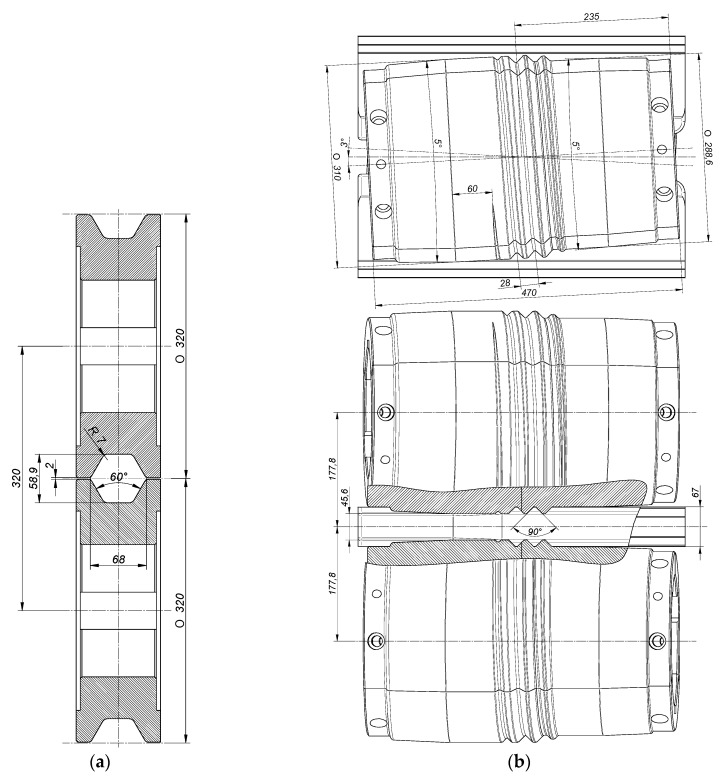
Design and configuration, with the dimension in mm: (**a**) grooved rolls for longitudinal rolling of a preform; (**b**) rolls with helical ribs for skew rolling of rods.

To show that the proposed technology could be used in the production of rods from scrap rail heads and to identify the potential limitations of this method, a comprehensive theoretical and experimental analysis of the rolling process was carried out. For the purposes of the study, we designed special tools for the two-stage rod rolling process. The rolls for the first stage of the process had the shape of cylinders with a grooved external profile. The cooperating grooves formed hexagonal passes. The basic geometric parameters of the groove pass used in the study are shown in Figure 2a. The second stage of rolling was performed using helical rolls. The shapes of the rolls along with the basic geometrical parameters are shown in Figure 2b.

A characteristic feature of the tools used in skew rolling is the helical ribs on their external profile, which taper into a smooth cylindrical surface in the sizing zone. This design is meant to make it easier for the tools to grip the irregularly shaped preform and to set it into rotational motion. The shape of the helical ribs was designed so that the volume of the stock material enclosed in the helical passes should remain constant regardless of the rotation angle of the rolls. The helical rolls were twisted relative to the rolling axis in opposite directions by the same angle, *γ* = 3°, which is equal to the helix angle. Additionally, two guides were used to hold the rolled material in the tool workspace. They formed an integral part of the tooling.

## 2. Finite Element Method (FEM) Analysis of Two-Stage Rolling of Rods from Scrap Rail Heads

**Figure 3:**
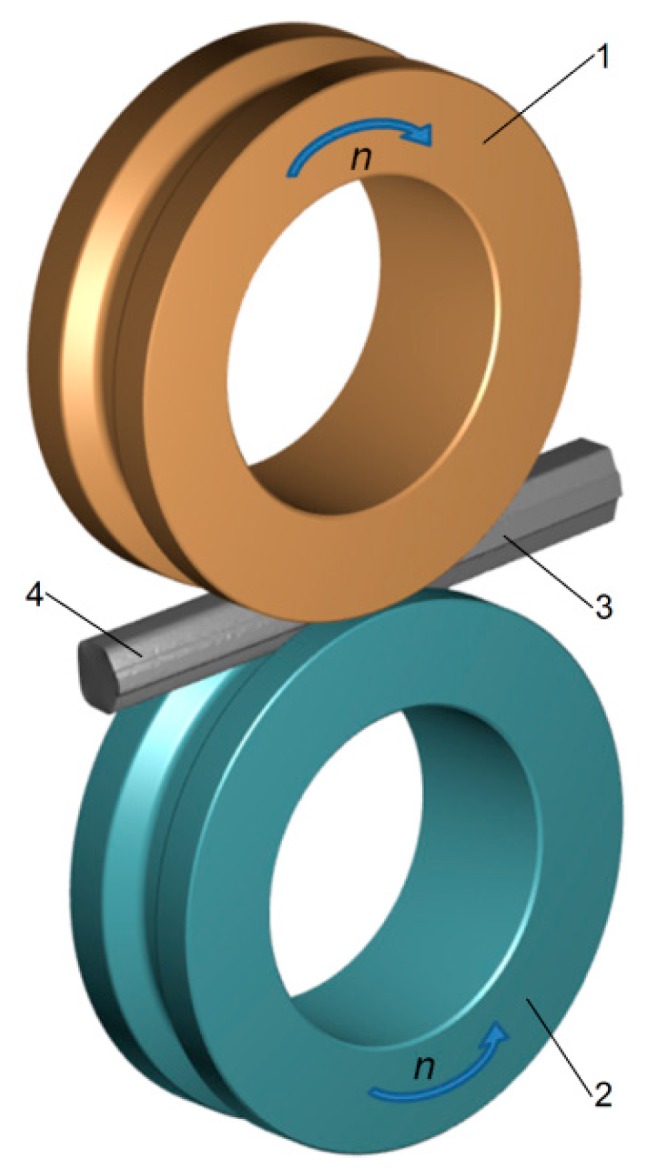
Geometric model of the first stage of the rolling process—longitudinal rolling in the grooved roll pass: 1,2—rolls, 3—rail head, and 4—preform for the second stage of rolling.

**Figure 4:**
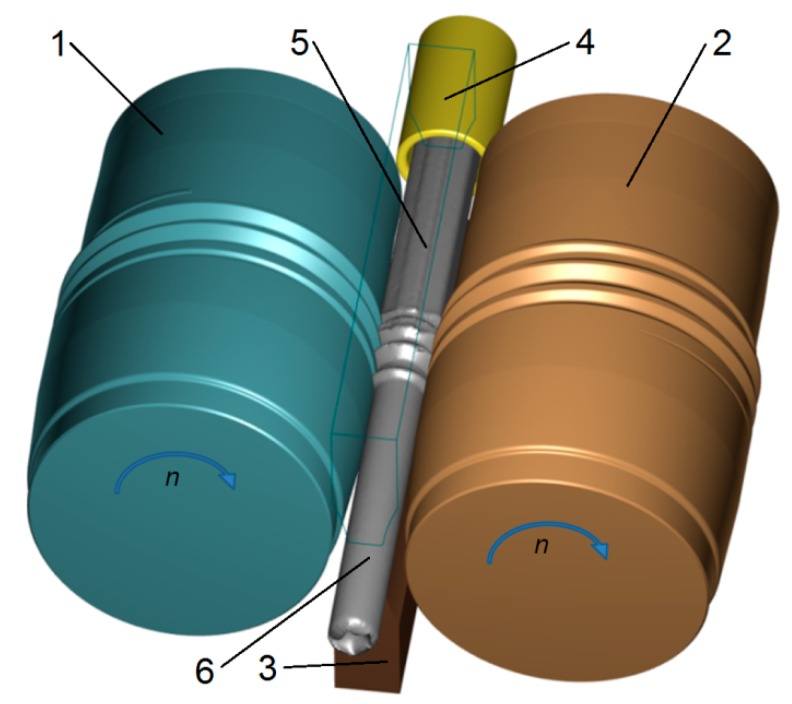
Geometric model of the second stage of the rolling process—skew rolling in the helical roll pass: 1,2—rolls, 3—guide, 4—guiding sleeve, 5—preform rolled in the first stage, and 6—final rod product.

Numerical simulations of the rod rolling process were carried out to verify the technological and structural assumptions adopted in the study. The simulations were performed using the finite element method (FEM). Calculations were made using FORGE NxT software under 3D deformation, taking into account thermal phenomena. During the calculations, the two-stage process of forming *ϕ* 45 mm diameter rods from the heads of scrapped S60 railway rails was analyzed. For the purposes of the study, two geometric models of the two stages of the rolling process were constructed (Figure 3 and Figure 4). The basic geometrical tool parameters (dimensions and positions) used in the calculations are given in Figure 2. The model of the first stage of rolling assumed that the rail head was compressed in the longitudinal rolling mill (Figure 3). This model featured two identical rolls (1 and 2) and a billet (3), which was rolled into a preform (4). The rolls had grooves machined around their circumference. Cooperating grooves were aligned to form a hexagonal pass. The billet used in the first stage of rolling was a partly worn head of an S60 rail with a length of *L* = 350 mm, cut off from the web at a height of 52.5 mm. The head was shaped so as to take account of the 15% allowable vertical and lateral rail wear limit. During the process, the rolls rotated in opposite directions at a constant speed of *n* = 30 rpm. The billet was modeled using first-order four-node tetrahedral elements, with the element size set to 1.0 mm. The initial temperature of the rail head was 1180 °C, and the temperature of the tools was constant throughout the process at 150 °C.

**Figure 5:**
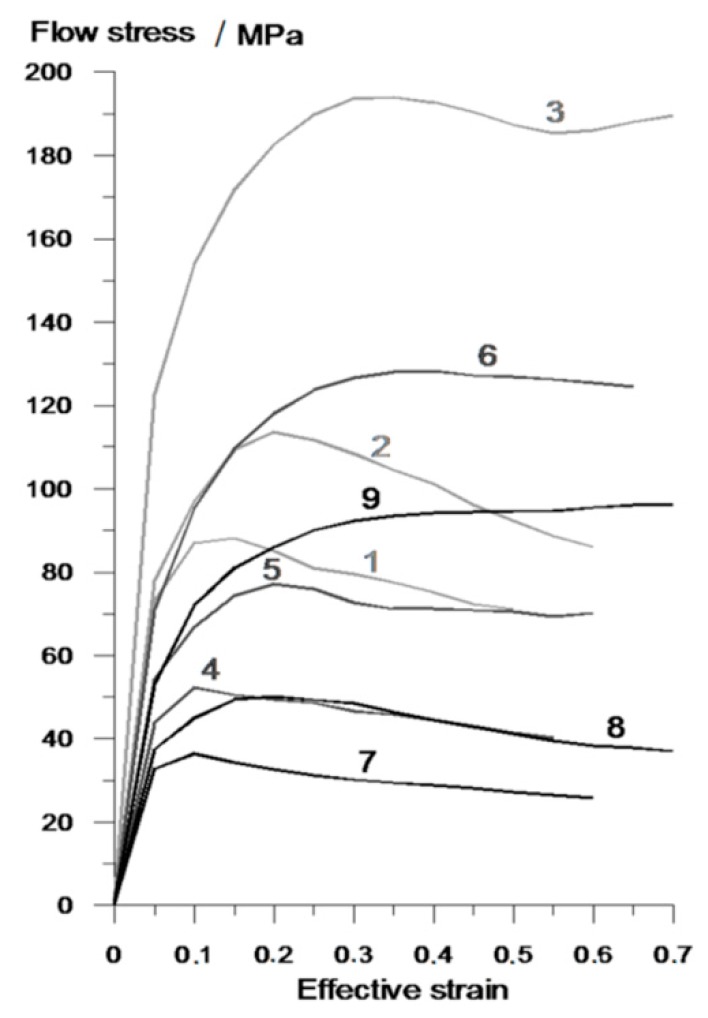
R200 steel flow curves used in finite element method (FEM) calculations: 1—*T* = 1000 °C, $\dot{\varepsilon }$ = 0.1 s^−1^; 2—*T* = 1000 °C, $\dot{\varepsilon }$ = 1 s^−1^; 3—*T* = 1000 °C, $\dot{\varepsilon }$ = 10 s^−1^; 4—*T* = 1100 °C, $\dot{\varepsilon }$ = 0.1 s^−1^; 5—*T* = 1100 °C, $\dot{\varepsilon }$ = 1 s^−1^; 6—*T* = 1100 °C, $\dot{\varepsilon }$ = 10 s^−1^; 7—*T* = 1200 °C, $\dot{\varepsilon }$ = 0.1 s^−1^; 8—*T* = 1200 °C, $\dot{\varepsilon }$ = 1 s^−1^; 9—*T* = 1200 °C, $\dot{\varepsilon }$ = 10 s^−1^.

The remaining parameters used in the calculations were: friction coefficient of the metal–tool contact surface *m* = 0.8 [24,25], heat exchange coefficient between metal and tool—5 kW/m^2^K—and between metal and the environment—0.35 kW/m^2^K. The material model of the stock material (R260 steel) from which the rails were made was based on literature data compiled by the present authors during previous plastometric tests [26]. The material characteristics, represented by flow curves, are shown in Figure 5. The preform formed in the first stage of the rolling process was transferred to the second stage along with the deformation and temperature histories (Figure 4). In the second stage, rods were rolled between two helical rolls (1 and 2). During the process, the rolls rotated in the same direction at the same speed *n* = 30 rpm. The blank was fed between the rolls through a guiding sleeve (4). During rolling, the material was held in the tool workspace by two guides (3). The remaining boundary conditions of the model were identical to those adopted for the first rolling stage. This allowed a comprehensive analysis of shape and temperature changes during the entire process of rolling a scrap rail head into rods.

**Figure 6:**
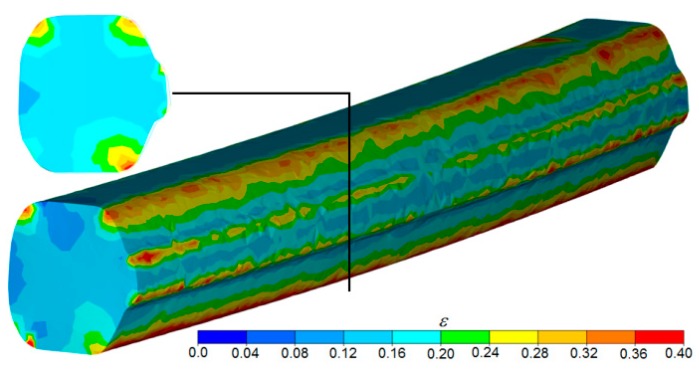
FEM strain distribution in the blank longitudinally rolled in the groove pass.

**Figure 7:**
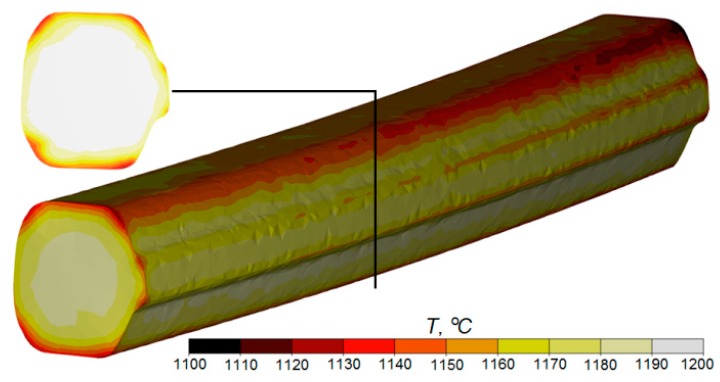
FEM temperature distribution in the blank longitudinally rolled in the groove pass.

The shape of the preform determined numerically in the rolling simulations and the distributions of effective strain and temperature are shown in Figure 6 and Figure 7. The images show that the rail head was only slightly compressed in the groove pass. The resulting blank had a cross-section whose convex corners can be circumscribed by a circle. This is important from the point of view of the second stage of rolling through helical passes. The low value of elongation in the groove pass is reflected in the distribution and magnitude of the strain rate. The preform was rolled in the longitudinal mill with an elongation coefficient of approximately *λ* = 1.15. As a result, the strains in the central part of the preform were uniform and small. Only near the corners of the preform could a local concentration of strains be observed. It was caused by slip of the material in the pass that occurred in areas where the material came in direct contact with tool surfaces. One of the most important parameters that affect the rolling process is temperature. As shown in Figure 7, the temperature of the preform after the first stage of rolling remained nearly as high as the initial temperature. Only the surface layers of the preform were cooler (due to the contact with the colder tools). It should be noted, however, that when the preform was transferred to the second rolling stage, the temperature evened out over the entire volume of the material, and the slight decrease in material temperature guarantees that the second stage of the process will proceed without interference.

**Figure 8:**
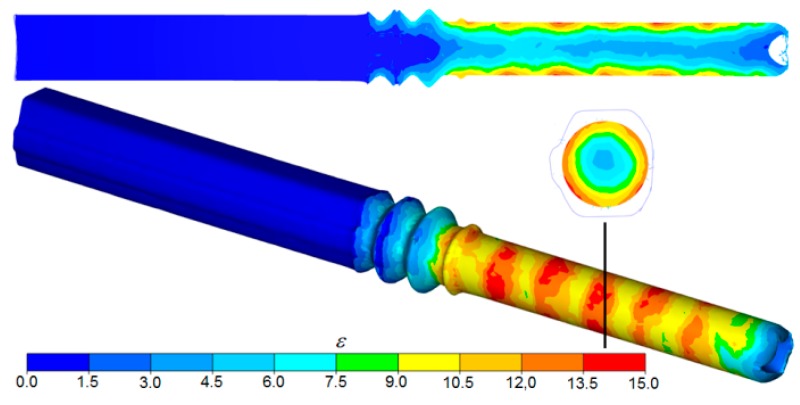
FEM strain distribution in a workpiece rolled in the helical pass of the skew rolling mill.

Interesting data come from the FEM simulation of the second stage of the process (rolling in helical passes). The helical ribs on the surfaces of the two rolls cut into the material to a depth corresponding to the adopted deformation, setting the workpiece in rotational motion and forming annular grooves on the circumference of the preform. The workpiece with the irregular outline was closed between cooperating grooves and was gradually deformed and elongated in the helical pass (with a decreasing depth) until it formed into a rod. The final shape of the rods was obtained as the cross-section of the workpiece was sized by the cylindrical surfaces of the tools located directly behind the helical pass. The kinematics of the process had a direct impact on the nature of strains in the material (Figure 8). As a result of the rotational compression of the material by the helical ribs on the rolls, strains concentrated in the surface layers of the rolled rod and then gradually decreased in the radial direction towards the rod axis. There were also visible ring-shaped strain concentration areas on the surface of the rod. The increase in strain was caused by the cyclic penetration of the helical collars into the material, during which annular grooves formed on the preform. In contrast, areas located between adjacent grooves were only deformed in the final coils of the pass, as a result of which strain in these zones had values that were several times lower. The differences in strain in the surface layers of the workpiece were also increased by friction forces, which caused circumferential displacement of the material and, thus, generated additional strain in the tangential direction.

**Figure 9:**
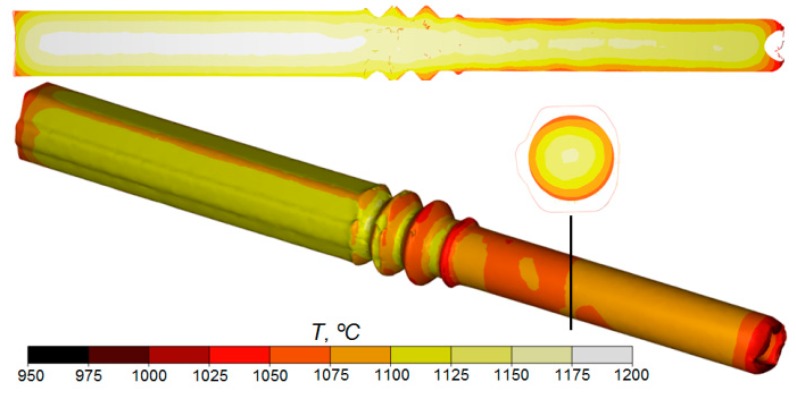
FEM temperature distribution in a workpiece rolled in the helical pass of the skew rolling mill.

During the numerical simulations, changes in the temperature of the workpiece were also analyzed, as shown in Figure 9. Temperature is a very important parameter of the process. It significantly affects plastic deformation resistance, effective strain, and the quality of the products obtained. A characteristic feature of the temperature distributions determined by FEM is their unevenness. While the temperature of the surface layers (in the range 100–150 °C) decreased, the central zone of the workpiece retained a high temperature (similar to the initial one). Obviously, the drops in the temperature of the outer layers of the material were caused by the dissipation of heat to the cooler tools and the surrounding environment. However, part of the heat loss was compensated for by the heat generated as a result of the conversion of plastic work and friction work into heat. As a result, the temperature of the rod after the second rolling stage was in the range of 1050–1100 °C (despite the relatively long duration of the process). This enables transition into the next stage of recycling of scrap rail heads (e.g., rolling of balls or other axisymmetrical forgings in a cross-wedge or a skew rolling mill) without the need to reheat the material.

One of the limitations of cross-wedge rolling and skew rolling is internal cracking of the material. The mechanisms of the formation of internal cracks are quite complex and are not yet fully understood. The internal cohesion of a workpiece may be destroyed by low-cycle fatigue of the material, caused by the cyclically changing nature of stress in its central zone of the material. In the analyzed process, the risk of material cracking was determined on the basis of the Cockroft–Latham ductile damage Equation (1):

(1) $$C=\int_{0}^{\varepsilon }\frac{\sigma_{1}}{\sigma_{i}}d\varepsilon ,$$

where: *C*—Cockcroft–Latham criterion, $\sigma_{1}$—maximum principal stress, $\sigma_{i}$—effective stress, and *ε*—effective strain.

**Figure 10:**
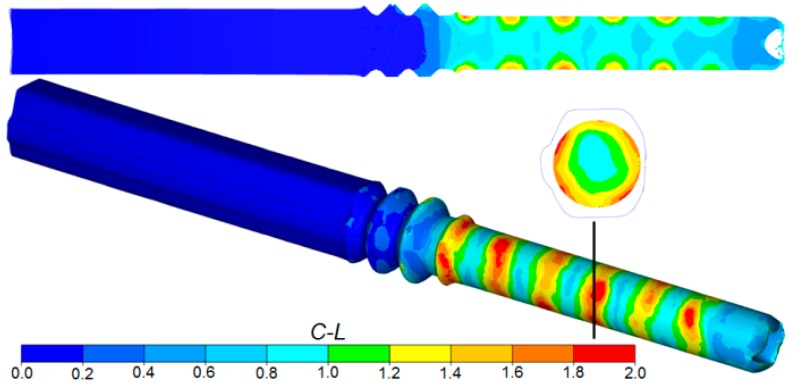
FEM distribution of the Cockroft–Latham damage criterion during rolling of rods in the helical pass of the skew rolling mill.

The critical values of the damage criterion were determined on the basis of tests of rotational compression of cylindrical specimens in a channel [27,28], the stress pattern of which corresponds to the stress distribution found in rotational forming processes. The results of the tests were used to determine the critical value of the damage criterion for R200 railway steel for a given temperature range. The determined value was *C* = 2.4 and was several times higher than that obtained in a tensile test [29]. The FEM distributions of the Cockroft–Latham criterion on the surface and in the axial and normal cross-sections of the rods are shown in Figure 10. The results indicated that there was no risk of internal cracking in the analyzed process. In the central zones of the rods, the values of the damage criterion were in the range of *C* = 0.7–0.9, which is three times less than the critical value at which cracks may form. Much higher values of the criterion (locally reaching the value of 2) were found in the surface layers, where they formed ring-like zones. These were the same areas where strain concentrations were observed. However, it should be noted that, also in this case, the values of the Cockroft–Latham criterion were below the critical value, and so there was no risk that the material will lose its cohesion during rolling.

## 3. Experimental Verification of Rod Rolling in Helical Passes

**Figure 11:**
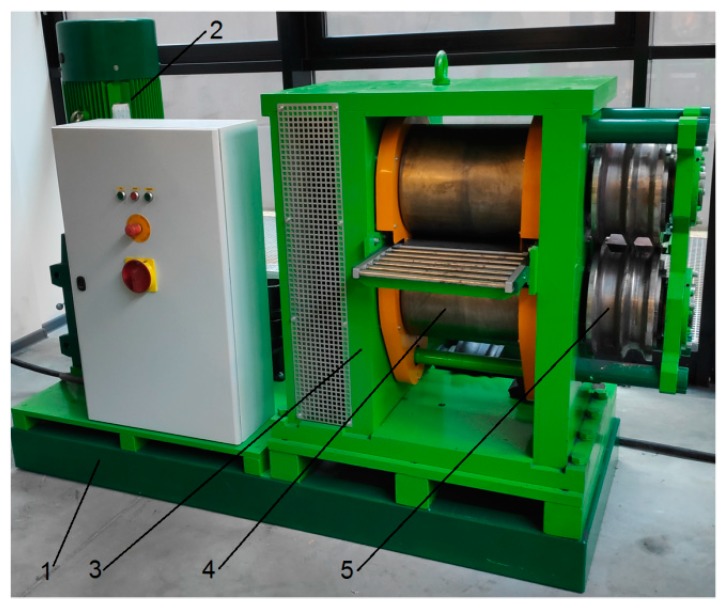
Console/frame longitudinal rolling mill: 1—frame, 2—drive unit, 3—rolling cage, 4—plain rolls, and 5—grooved rolls.

**Figure 12:**
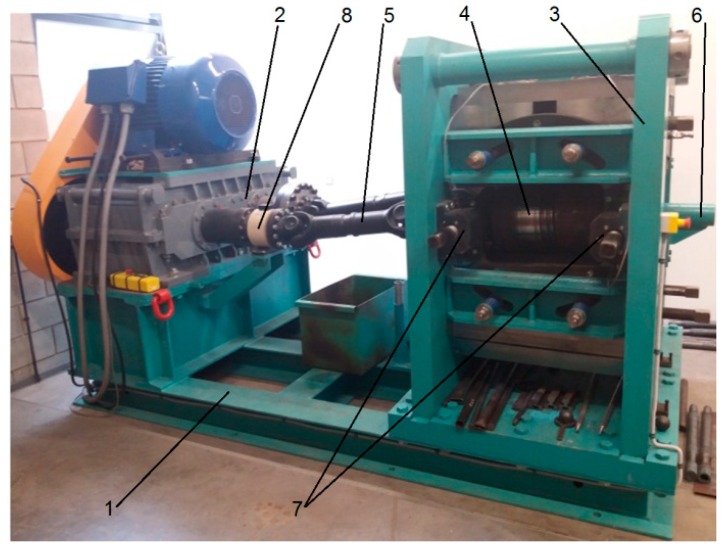
Skew rolling mill: 1—frame, 2—drive unit, 3—rolling cage, 4—working roll, 5—jointed shafts, 6—guiding sleeve, 7—mounting brackets for pressure sensors, and 8—torque sensor.

Preform rolling tests were carried out in a longitudinal rolling mill [30]. Rods, on the other hand, were rolled in a skew rolling mill [31]. Both rolling mills had been designed at the Lublin University of Technology and are currently installed at the Center of Innovative Technologies of the Lublin University of Technology. The longitudinal rolling mill used in the tests was a frame–console mill (Figure 11). The structure of the mill allows it to be used for longitudinal rolling of sheets, strips, foils and flat bars, which are produced using plain roll barrels (frame configuration). The console configuration can be used for metallurgical groove rolling and forge rolling on periodically changing segments. The basic assembly of this mill included a frame, an electric engine with a helical-bevel gear motor, and a rolling cage with an eccentric roll-axis-spacing adjustment system for each roll. In addition, the rolling mill was equipped with a system for recording torque during the rolling process. Preform rolling tests were performed using grooved rolls positioned in the console configuration. Tests of the second stage of rolling were performed using a skew rolling mill with a segmented design (Figure 12). The machine consisted of a support frame, a drive system, a rolling cage, and a drive train. The rolling mill was also equipped with sensors for measuring the torque and thrust force of the tools; these devices cooperate with the machine's control system and protect the drive system and the tools against overload and damage. The basic technological parameters of both rolling mills are given in Table 2.

**Figure 13:**
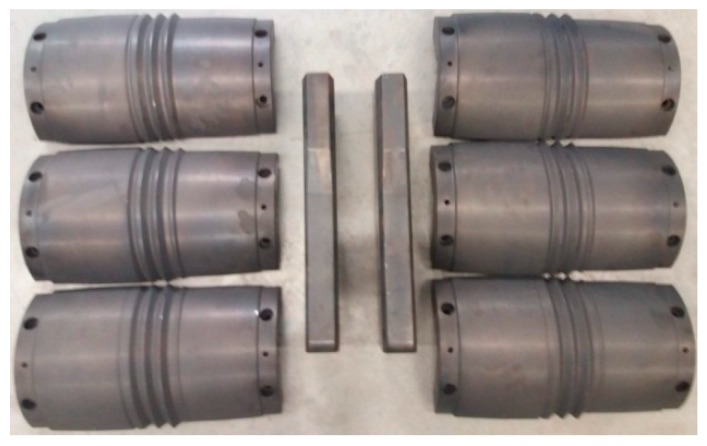
A set of segmented tools with helical passes for rolling rods from scrap rail heads.

For the purposes of the experiments, the grooved rolls forming a hexagonal pass were mounted on the console shafts of the longitudinal rolling mill. Each roll consisted of two segments, which were mounted on the perimeter of the outstretching shafts using two lock nuts. The rolls with the helical ribs were mounted on the working shafts of the skew rolling mill (Figure 13). Each tool set consisted of three individual segments, mounted every 120° on the perimeter of the shafts. In addition, two (upper and lower) guides were installed between the rollers to hold the rolled material in the tool workspace. The set of tools consisting of two segmented rolls and two guides used in the tests of the second stage of the rolling process is shown in Figure 13. The geometric parameters of the tools and their positions were consistent with the specifications given in Figure 2.

**Figure 14:**
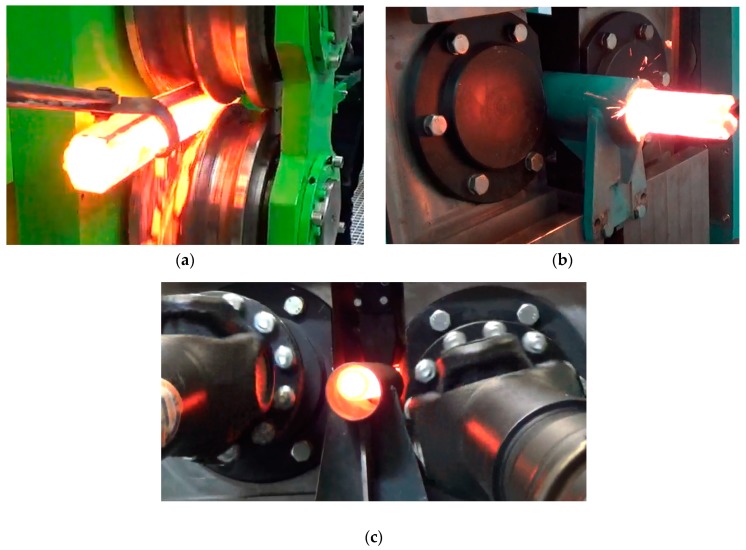
The sequential stages of rolling of rods from scrap rail heads: (**a**) rolling of the preform in the longitudinal mill; (**b**) feeding of the rolled preform to the skew mill; (**c**) a rod rolled in a helical pass, leaving the work space of the skew mill.

Before processing, blanks (350 mm long rail head sections cut off from worn railway rails) had been heated in an electric chamber furnace to a forming temperature of 1180 °C and were then rolled longitudinally in the hexagonal pass (Figure 14a). Next, the rolled preforms were transferred to the skew rolling mill and fed into its workspace (Figure 14b). During rolling, the billet was gripped by the wedge collars of the rotating rolls, which set it in a rotary motion and pulled it into the helical passes formed by the cooperating grooves and collars of the two rolls. As the working collars of the rolls pressed against the preform, its irregular cross-section gradually changed into a circular cross section, and a rod was formed. The rods were automatically removed from the working space of the skew rolling mill through the discharge sleeve (Figure 14c) located behind the rolling cage. 

**Figure 15:**
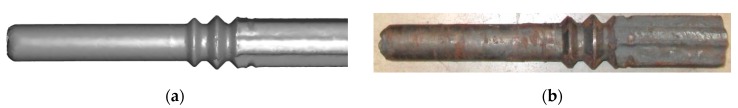
Changes in the shape of the workpiece during skew rolling of preforms obtained from scrap rail heads: (**a**) determined using FEM; (**b**) determined experimentally.

The kinematics of material flow during the first stage of rolling in the groove pass are relatively simple. The material enclosed within the grooves of the two rolls is gradually compressed by the tools which rotate in opposite directions. As a result, the cross-section of the rail head is reduced, and a preform is formed in which the height of the cross-section is similar to the width. A much more complex nature of material flow is found in the second stage of rolling in the helical pass. In order to determine the kinematics of material flow, the process of skew rolling of rods from preforms rolled from scrap rail heads was halted in the steady state phase (Figure 15). The images clearly show that the irregular cross-section of the preform changed into a circular one in the helical pass. The rolls first formed annular grooves in the preform to a depth that corresponded to the diameter of the finished rod product, and then during three subsequent rotations of the tools, the helical collars on the rolls further reduced the cross-section of the workpiece until a rod was obtained.

**Figure 16:**
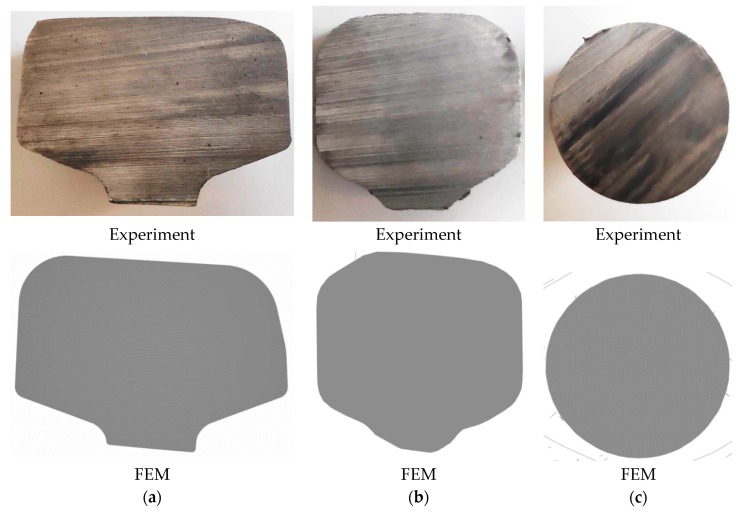
Changes in the cross-sectional shape of the rail head during rod rolling: (**a**) head cut off from a scrapped rail; (**b**) preform formed in the hexagonal pass of the longitudinal mill; (**c**) rod rolled in a helical pass of the skew mill.

A comparison of the cross-sections of the semifinished products obtained in the numerical simulation (Figure 15) and those rolled in the sequential stages of the rolling process (Figure 16) shows that the computational and experimental results are in a quite good accordance. Moreover, the workpieces were inspected after each of the rolling stages, and then the preforms and the rods were measured. The results of the measurements are given in Table 3. The inspections and measurements showed that the products were of satisfactory quality and precision typical of hot-formed products. The surface of the rolled rods was clean and free of scale. Also, no lapping was observed despite the irregular shape of the preform.

**Figure 17:**
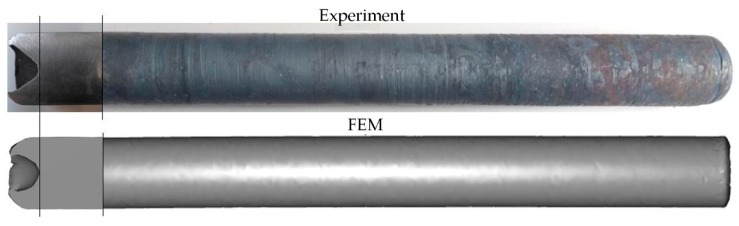
Shape of the rods after the second stage of rolling, with cavities visible on the end faces.

However, small protrusions, with the shape of a helical line, were found on the surfaces formed in the last stage of rolling. The protrusions were 0.1–0.2 mm high and fell within the permissible range of deviation for hot-rolled metallurgical products (which is *ϕ* $45_{-0.4}^{+0.6}$ mm for products in this diameter range [32]). In addition, quite deep cavities (Figure 17), caused by surface flow of the material, were observed on the end faces of rods, both those rolled in the experiments and those modelled numerically. It should be noted, however, that formation of cavities is a characteristic feature of rotational metalworking processes, which is why the extreme ends of rods produced in those processes are treated as stock allowance. The good agreement between the experimental and numerical results, both quantitative (the observed differences in size did not exceed 1%) and qualitative (the shapes of the semi-finished product and the finished product obtained in the experiments were consistent with the numerically determined outline) (Figure 15, Figure 16 and Figure 17), confirmed the accuracy of the design and technological assumptions adopted in the experiments and the correctness of the numerical models. In turn, the quality of the products indicates that the proposed technology can be effectively used for rolling rods from worn rail heads.

**Figure 18:**
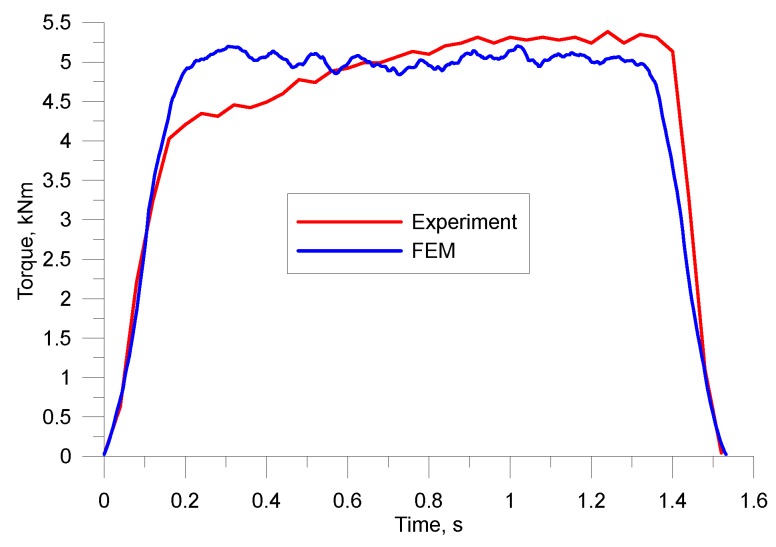
Distribution of torques during longitudinal rolling of preforms from scrap rail heads.

During the tests, force parameters of the process were also registered. More precisely, torques were recorded for longitudinal rolling and radial forces, and rolling torques were registered for skew rolling. Torque curves for longitudinal rolling in the hexagonal pass are shown in Figure 18. The figure shows that the quantitative agreement between the experimental and numerical results was quite good. The maximum torques in both cases slightly exceeded 5 kNm. However, there was a visible difference in the shapes of the two functions. In the case of the numerical model, the torque remained constant at all times, but during the experiment it gradually increased. The difference may be caused by the drop in the temperature of the material during the experiment, which affects the value of plastic deformation resistance.

**Figure 19:**
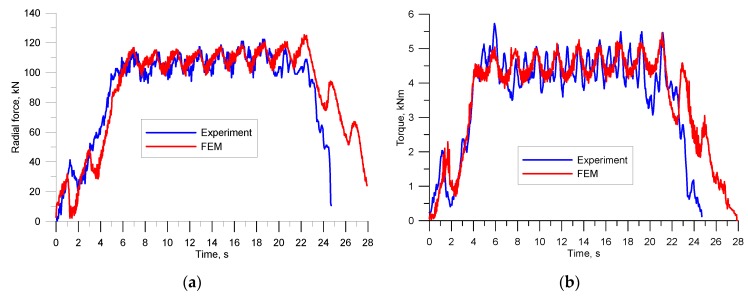
Distribution of force parameters during rolling of rods in the skew rolling mill: (**a**) radial force; (**b**) torque.

The distributions of force parameters for the second rolling stage are shown in Figure 19. Figure 19a presents the radial force curves, and Figure 19b shows torque curves. A characteristic feature of these distributions is their oscillating shape associated with the cyclic penetration of the wedge collars into the material every one full rotation of the rolls. The results of the FEM simulations and the results of the measurements made during the experimental tests show a fairly good agreement, both quantitative (mainly in terms of mean values) and qualitative (similar nature of changes in forces and moments). The main difference between the experimental and numerical results is related to the size of the amplitude of the oscillation of forces and moments. The numerically determined curves were characterized by smaller force and moment oscillation amplitudes. This may be related to the simplifications adopted in the calculations. The numerical models were developed under the assumption of perfect rigidity of the tools. In reality, however, the components of the rolling mill and the tools themselves bend elastically, which affects the shapes of the force and torque curves by increasing the amplitude of oscillation of these values. Also worth noting are the maximum and minimum values of the forces and moments, which, in real settings, determine whether the process can or cannot be performed in industrial conditions. It can be seen that the maximum torques were relatively small at 5 kNm, a value that can be obtained in commercially available skew rolling mills. The extreme values of radial forces have an impact on the value of elastic deformation of the mill and, hence, on the precision of rolled rods. In the analyzed process, the maximum force values are also small; they did not exceed 130 kN, which means they will not lead to excessive elastic deformation of the mill body and deflection of the rolls.

## 4. Conclusions

In this article, we proposed an innovative method of rolling rods from heads cut off from scrap rails. Currently, worn rails are reprocessed mainly using metallurgical processes; however, the analysis conducted in the present study indicates that recycling of some elements of a rail using rolling technologies can yield measurable benefits. The results of the theoretical and experimental investigations clearly show that rods can be rolled from scrap rail heads in two stages. In the first stage, a rail head is compressed in a longitudinal rolling mill to obtain a preform, which in the second stage is rolled into rods in helical passes of a skew rolling mill. Compared to the traditional process of longitudinal rolling in groove passes, the proposed solution significantly reduces the duration of the process and the costs of its execution. Of no small significance is also the fact that the new technology allows to roll rods of different diameters using one set of tools. The diameter of the rolled rod depends solely on the setting of the skew rolling mill, and not on the geometry of the rolls used. The traditional groove rolling requires calibration and the use of different rollers to obtain different rod diameters. Furthermore, the kinematics of the skew rolling process makes it possible to obtain rods that are much less ovalized and much straighter compared to those produced by longitudinal rolling. These properties allow the rail reprocessing technology to be applied in industry, not only by steelworks and rolling mill plants, but also by small forging shops producing semifinished products for their own use (e.g., as billets for forging or forge rolling). The design and technological solution proposed in this study allows not only to utilize and effectively reprocess scrap rail heads, but the process of two-stage rolling can also be used to form rods from the other parts of worn rails (the web and the foot), thus allowing for complete recycling of railway scrap without the need to use metallurgical processes. The results of the study lead to the following conclusions:

- Rods can be efficiently rolled from scrap rail heads in two passes in a process that combines longitudinal and skew rolling.
- The preform rolled from a scrap rail head in the longitudinal rolling mill can be used to roll rods with a wide range of diameters in one (skew) rolling mill and with one set of helical tools.
- The strains that develop during the rolling of rods in the helical passes are unevenly distributed.
- Despite a nonuniform cross-sectional outline of scrap rail head, the products rolled in the skew rolling mill are characterized by a high quality and precision.
- During skew rolling of rods, cavities form on the end faces of the rods; they are treated as stock allowance.
- Rolling in helical passes is performed at relatively low radial forces and torques.
- The good agreement between the experimental and simulation results supports the use of numerical modelling in the analysis of complex metalworking processes.
- Further research should be carried out to determine the impact of technological parameters on the course of the rolling process and the quality of the products obtained.

## Figures and Tables

**Table 1:** Geometric parameters and elongation coefficients for the successive stages of the rolling process.

Stage	Rail Head	Worn Rail Head	Preform	Rod
Cross-sectional area *S*, mm^2^	3050	2800	2400	1635
Elongation coefficient *λ* = *S*_0_/*S*_1_	-	-	1.16	1.45

**Table 2:** Basic parameters of the rolling mills used in the rod rolling tests.

Parameter/Rolling Mill Type	Skew Rolling Mill	Longitudinal Rolling Mill
Position of rolls in the working cage; mm	horizontal	vertical
Nominal roll diameter; mm	320	320
Working length of roll barrel; mm	400	320/160
Minimum distance between roll axes; mm	300	310
Maximum distance between roll axes; mm	350	330
Possible inclination of the shaft axis to the rolling axis; deg	±12	-
Minimum rotational speed of rolls; rpm	15	-
Minimum rotational speed of rolls; rpm	30	30
Nominal torque on one roll (for 15 rpm); kNm	20	-
Nominal torque on one roll (for 30 rpm); kNm	10	5
Machine dimensions; m	3.2 × 1.8 × 2.1	2.4 × 1.5 × 1.4
Machine weight; kg	17,500	4200
Motor power; kW	60/80	22

**Table 3:** Geometric parameters of rolled rods and preforms.

Research Method	H; mm	B; mm	h; mm	b; mm	d; mm
FEM	73.9	52.5	58.9	57.6	45.1
Experiment	73.9	53.0	59.2	58.0	45.3